# Cost-effectiveness Analysis of Fluorouracil, Leucovorin, and Irinotecan versus Epirubicin, Cisplatin, and Capecitabine in Patients with Advanced Gastric Adenocarcinoma

**DOI:** 10.1038/srep36060

**Published:** 2016-11-08

**Authors:** Feng Wen, Hanrui Zheng, Yifan Wu, John Wheeler, Xiaoxi Zeng, Ping Fu, Qiu Li

**Affiliations:** 1 Department of Medical Oncology, Cancer Center, State Key Laboratory of Biotherapy, West China Hospital, Sichuan University, China; 2 West China Biostatistics and Cost-Benefit Analysis Center, Sichuan University, China; 3 Department of Clinical Pharmacy, West China Hospital, Sichuan University, China; 4 School of Public Health, University of Michigan, Ann Arbor, Michigan 48109, USA; 5 Division of Nephrology of Department of Internal Medicine, West China Hospital, Sichuan University, China

## Abstract

No standard treatment has been accepted widely for the first-/second-line therapy for advanced gastric cancer (AGC). The current study aimed to determine a preferred strategy between FOLFIRI (fluorouracil, leucovorin, and irinotecan) and ECX (epirubicin, cisplatin,and capecitabine) for AGC from the cost-effectiveness perspective. According to a French intergroup study, two groups (ECX arm and FOLFIRI arm) and three health states (progression-free survival (PFS), progressive disease (PD) and death) were analyzed in the current Markov model. All the medical costs were calculated from a Chinese societal perspective. Although FOLFIRI was an acceptable first-line therapy in the treatment of AGC with a better time-to treatment failure (TTF) compared to ECX, ECX arm (ECX followed by FOLFIRI) gained 0.08 quality-adjusted life months (QALMs) more effectiveness benefit compared with FOLFIRI arm (FOLFIRI followed by ECX). Additionally, a lower cost was found in ECX arm ($23,813.13 versus $24,983.70). Hence, the strategy of FOLFIRI arm is dominated by ECX arm ($4,125.8 per QALM in FOLIRI arm; $3,879.724 per QALM in ECX arm). ECX followed by FOLFIRI was a preferred strategy with more effectiveness and lower cost compared with FOLFIRI followed by ECX for the treatment of AGC.

Though the incidence and mortality rates of gastric cancer (GC) have decreased from a global perspective, it still ranks second in cancer-related deaths annually^1^. More than half of the newly diagnosed cases happen in developing countries and about 70% of deaths occur in the less developed areas. Notably, approximately 35–42% of cases all over the world occur in China^2^. However, the clinical outcomes of advanced gastric cancer (AGC) is still frustrating, with a median overall survival (OS) of less than five months under the management of best supportive care^3^.

Generally, most patients suffer from disease recurrence or metastasis after surgeries^4^. Palliative chemotherapy for AGC patients has achieved a significant development in improving the quality of life (QOL) and prolonging the OS^3^. However, no standard chemotherapy regimens have been widely accepted as the first-line or second-line treatment in clinical practice^4^. In the latest decade, the appearance of some new regimens, including oral use of fluorouracil, oxaliplatin, irinotecan and docetaxel are promising for this indication. New combinations, for instance, epirubicin, cisplatin and capecitabine (ECX), epirubicin, oxaliplatinand capecitabine (EOX), fluorouracil, leucovorin and oxaliplatin (FOLFOX) as well as docetaxel, cisplatin and fluorouracil (DCF) have been proven to be equivalent or superior compared with previous combined regimens, such as cisplatin and fluorouracil (CF) and epirubicin, cisplatin, and fluorouracil (ECF) as the first-line treatment of AGC, which are widely used in practice currently; however, the overall survival is still less than one year^5^,^6^,^7^. Furthermore, salvage chemotherapy as the second-line treatment of AGC has been well researched in some clinical trials^8^. Among all the regimens, irinotecan is a promising semi-synthetic anti-cancer drug derived from camptothecin. In the V306 study, combinations of irinotecan and fluorouracil (IF) had a better tolerability and reactivity (32% vs 26%) than CF^9^. Additionally, irinotecan, fluorouracil and leucovorin (FOLFIRI) gained more response rate (40%,13% and 27% respectively) and longer time to progression (TTP) (6.0 months, 3.2 months and 4.9 months respectively) as well as a higher OS (11.0 months, 6.8 months and 9.5 months respectively) compared with LF (fluorouracil and leucovorin) and CLF (cisplatin, fluorouracil and leucovorin) in a phase II clinical study^10^. Based on available data, FOLFIRI is a considerable option for patients with AGC^11^.

In a recent open labeled, prospective, randomized phase III clinical trial conducted by a French intergroup among 71 French centers, FOLFIRI versus ECX as a first-line treatment for AGC patients was studied. The results demonstrated that patients in FOLFIRI arm had a significant longer time to treatment failure (TTF) compared to ECX as a first-line treatment for AGC. Nevertheless, no statistical differences were found in progression-free survival (PFS) and OS^12^. As a non-platinum-based chemotherapy alternative, FOLFIRI clearly appears to be a great potential candidate for patients who are unable to tolerate platinum-involved regimens. Besides, FOLFIRI is recommended as an acceptable combination in the first-line treatment of AGC by national comprehensive cancer network (NCCN) clinical practice guidelines. It is also regarded as a promising backbone for the target therapy, however, the medical burden would be increased dramatically with the combination of chemotherapy and target agents. Therefore, it is of great importance to make a preferred decision for AGC patients in the first-line and second-line treatments.

A more careful decision ought to be made in developing countries, such as China. Treatments combinatorial optimization from the perspective of cost-effectiveness should be under serious consideration for decision-makers to maximize the clinical effectiveness with reasonable medical costs. Therefore, the aim of this study is to to determine the preferred strategy between FOLFIRI followed by ECX and ECX followed by FOLFIRI for AGC patients with a cost-effectiveness analysis based on the French intergroup study from the Chinese societal perspective.

## Results

### Effectiveness

**Figure 1:**
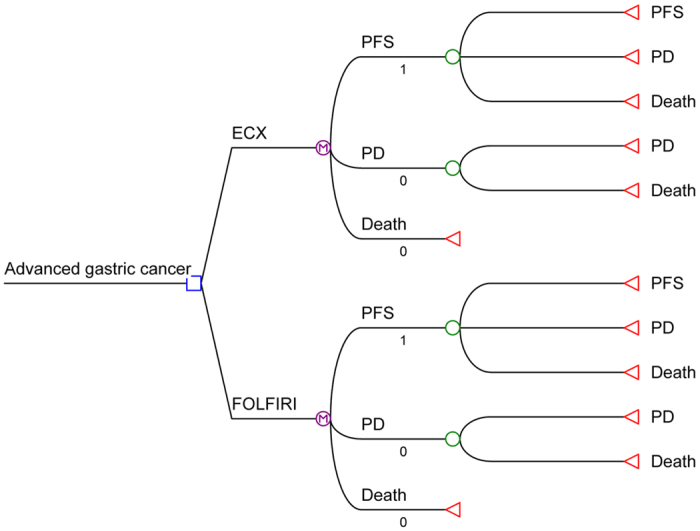
Markov model for advanced gastric cancer. According to the study profile, two groups were analyzed: group ECX, patients with advanced gastric cancer treated with ECX followed by FOLFIRI; group FOLFIRI, patients with advanced gastric cancer treated with FOLFIRI followed by ECX. A Markov model comprising three health states (progression-free survival, progressive disease and death) was built. ECX, epirubicin, cisplatin, and capecitabine; FOLFIRI, fluorouracil, leucovorin, andirinotecan; PD, progression disease; PFS, progression-free survival.

According to the results of the French group study, the difference of survival benefit was not significant between the ECX and the FOLFIRI groups, but the PFS and OS were slightly longer in FOLFIRI arm than in ECX arm(PFS: 5.8 months versus 5.3 months; OS: 9.7 months versus 9.5 months). Based on the present model result (Fig. 1), the ECX arm gained 6.14 QALMs, while the FOLFIRI gained 6.06 QALMs. In detail, effectiveness for PFS state was 3.69 QALMs for ECX arm and 3.93 QALMs for FOLFIRI arm, and effectiveness for PD state was 2.45 QALMs for ECX arm and was 2.12 QALMs for FOLFIRI arm. The details are listed in Table 1.

### Costs

Treatment associated costs were stated as per month per patient in one transition cycle from the Chinese societal perspective in 2015, which are listed in Table 2. Since FOLFIRI regimen cost much more than ECX regimen ($2,275.46 versus $959.68), cost for PFS state was higher in FOLFIRI arm than in ECX arm ($18,481.71 versus $8,499.92). On the contrary, cost for PD state was higher in ECX arm than in FOLFIRI arm ($15,313.21 versus $6,501.98). In total, the incremental cost was $1,170.57 for FOLFIRI arm compared with ECX arm ($24,983.70 versus $23,813.13). Information regarding 3/4 AEs analyzed in the model is illustrated in Table 2, and the costs of AEs prevention or treatment were considerably cheaper compared with chemotherapy agents in general.

### Cost-effectiveness analysis

**Figure 2:**
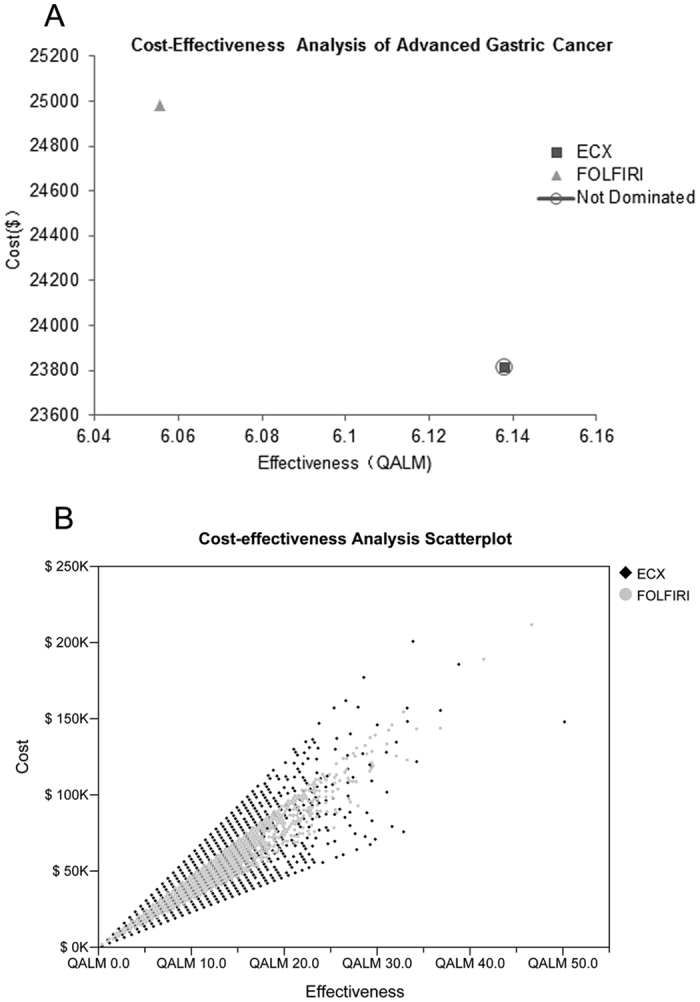
Cost-effectiveness pictured with two groups. (**A**) Two groups were analyzed: group ECX, patients with advanced gastric cancer treated with ECX followed by FOLFIRI; group FOLFIRI, patients with advanced gastric cancer treated with FOLFIRI followed by ECX. (**B**) Cost-effectiveness scatterplot of two groups. ECX, epirubicin, cisplatin, and capecitabine; FOLFIRI, fluorouracil, leucovorin, andirinotecan; QALM, quality-adjusted life-month; CE, Cost-effectiveness.

Obviously, ECX followed by FOLFIRI strategy was more effective with less cost compared with FOLFIRI group. Namely, the ECX group spent $3,879.72 per QALM compared with $4,125.84 per QALM for FOLFIRI group (Fig. 2A). Generally, most cost/effectiveness scatterplot of FOLFIRI group are distributed in the middle of ECX group (Fig. 2B).

### Sensitivity analysis

**Figure 3:**
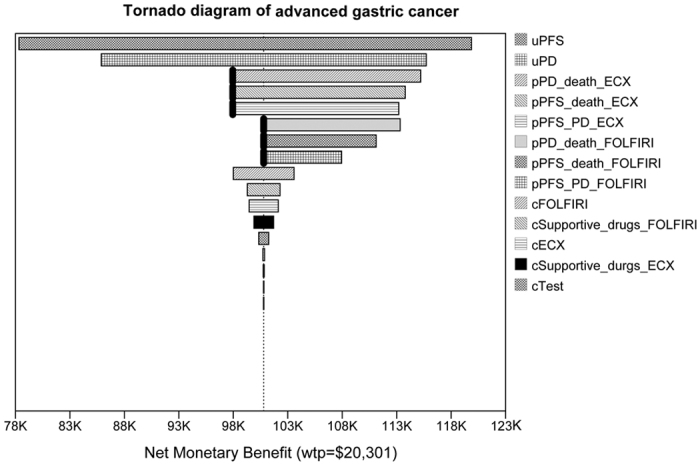
Tornado diagram of one-way sensitivity analysis. Tornado diagram summarized the results of one-way sensitivity analysis to identify model variables associated with the two strategies in the treatment of advanced gastric cancer. The influential factors were listed descending with the variation of value. ECX, epirubicin, cisplatin, and capecitabine; FOLFIRI, fluorouracil, leucovorin, andirinotecan; AE, adverse event; K, a thousand times; pPD_death_ECX, transition probability for PD state to death in ECX arm;pPFS_death_ECX, transition probability for PFS state to death in ECX arm; pPFS_PD_ECX,transition probability for PFS state to PD state in ECX arm; pPD_death_FOLFIRI, transition probability for PD state to death in FOLFIRI arm; pPFS_death_FOLFIRI, transition probability for PFS state to death in FOLFIRI arm; pPFS_PD_FOLFIRI, transition probability for PFS state to PD state in FOLFIRI arm; cFOLFIRI, FOLFIRI cost; cSupportive_FOLFIRI, supportive cost in FOLFIRI arm; cECX, cost for ECX; cSupportive_ECX, cost for supportive drugs for ECX arm.

In order to test the responsiveness of the model and the robustness of our results, one-way sensitivity analysis was conducted. The variables in the sensitivity analysis varied at a range of ±30%, and the results are shown in the tornado diagram in Fig. 3. Net monetary benefit (NMB) was applied in the tornado diagram. According the calculation formula *NMB* = *Effectiveness* × *WTP* − *Cost,* NMB combines cost, effectiveness and WTP into a single measurement, and it can show at what point in variable range do we have a change in the recommended strategy based on cost‐effectiveness(threshold)^13^.

Results of the sensitivity analysis depicted that the utilities for PD state and PFS state were the most influential parameters, followed by transition probabilities in ECX arm and FOLFIRI arm. Specifically, with the value changes of PD state utility and PFS state utility, the preferred option always was ECX arm, and the rang of NMB for ECX arm were obvious, which were demonstrated in the tornado diagram. In terms of transition probabilities for PD state to death (pPD_death), PFS state to death (pPFS_death) and PFS state to PD state (pPFS_PD) in both groups, the recommended strategy changed with the variation of values. For instance, when the value of pPD_death for ECX arm (pPD_death_ECX) arrived at 0.17, the preferred decision changed from ECX to FOLFIRI. Of note, changes for FOLFIRI cost (cFOLFIRI) had the greatest impact on the results among all the treatment related costs, followed by the supportive cost in FOLFIRI arm (cSupportive_FOLFIRI). Additionally, the cost-effectiveness analysis was also sensitive to the cost for ECX (cECX) and cost for supportive drugs for ECX arm (cSupportive_ECX).

### Probabilistic sensitivity analysis (acceptability frontier)

**Figure 4:**
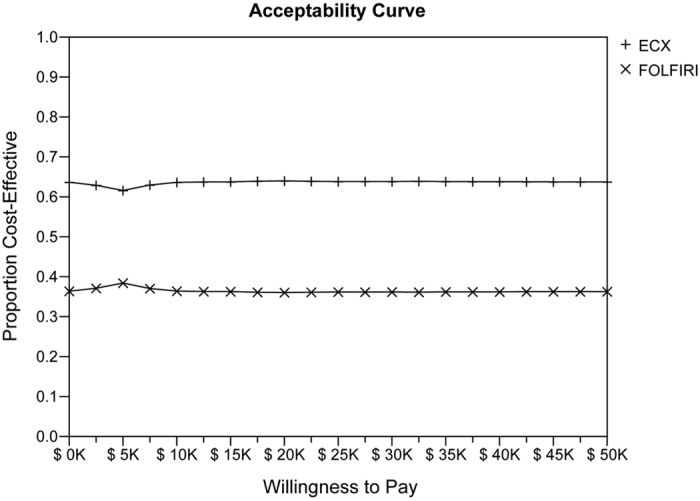
Probabilistic sensitivity analysis (acceptability frontier). The cost-effectiveness acceptability frontier shows the probabilistic sensitivity analysis -based probability of strategies being cost-effective in two strategies. For different willingness to pay thresholds, the proportion for ECX group was stably above 60%, while the FOLFIRI group was below 40%. K, a thousand times; ECX, epirubicin, cisplatin, and capecitabine; FOLFIRI, fluorouracil, leucovorin, andirinotecan.

The cost-effectiveness acceptability frontier showed the probabilistic sensitivity analysis-based probability of strategies being cost-effective. For different WTP thresholds, different strategies are optimal. With the respect to WTP, as the value varied from $0 to $100,000, the acceptable proportion of ECX group was stably above 60%, while the acceptable percentage for FOLFIRI group was stably below 40% (Fig. 4).

## Discussion

The prognosis of AGC is disappointing, even though progression has been made with the approvals of several new drugs and different therapeutic combinations. However, the 5-year survival rate still remains less than 20%^1^. As we know, efficacy development always remains of paramount importance for the new agent evaluation in the AGC treatment researches. At the same time, a low rate of toxicity profile as well as convenience of administration are also two important considerations^14^.

Until now, several molecular targeted agents for the treatment of AGC have been studied. Among them, anti-epidermal growth factor receptor (EGFR) and anti-vascular endothelial growth factor receptor (VEGFR) regimens failed to achieve treatment efficacy in the several clinical trials, except for anti-HER2 (herceptin)^15^,^16^,^17^. As the effective targeted therapy for AGC, herceptin has been shown to prolong the overall survival in a specific population with HER2 over expression^18^. Moreover, for patients without HER2 overexpression, chemotherapy with two or three cytotoxic regimens plays important role in the palliative setting. As a consequence, the medical burden dramatically increased, especially for new-targeted drugs candidates in clinical practice. Therefore, it is urgent to choose a better treatment combination as well as the best sequence among the available therapeutic strategies to optimize the OS of AGC and to improve the QOL from the perspective of cost-effectiveness analysis.

The French group study was designed to compare the efficacy of ECX and FOLFIRI in the first-line treatment of AGC, and the regimens of second-line were predefined (FOLFIRI for the ECX group and ECX for the FOLFIRI group)^12^. The outcome indicated that FOLFIRI was an acceptable regimen in the first-line treatment of AGC, but the survival benefit was not significant. Finally, it is necessary to figure out the preferred strategy not only from the angle of clinical outcome but also in the context of medical costs.

Even though FOLFIRI was regarded as an acceptable first-line therapy for AGC with a better TTF compared with ECX in the French study, ECX followed by FOLFIRI (ECX arm) was estimated to be more beneficial with gaining 0.08 more QALMs compared with FOLFIRI followed by ECX (FOLFIRI arm) in the current research. Additionally, a lower cost was found in ECX arm ($23,813.13 versus $24,938.70). Additionally, the strategy of FOLFIR arm is dominated by ECX arm with a cost of $3,879.72 per QALM for FOLIRI arm compared with $4,125.84 per QALM for ECX arm. With the respect to WTP, as the value varied from $0 to $100,000, the acceptable proportion of ECX followed by FOLFIRI group was stably more than 60%, which was always about 20% more than FOLFIRI followed by ECX group.

Although the chemotherapy regimens for AGC have been widely studied and extensively used in clinical practice, only limited number of research have been conducted from an economic perspective^14^. Compared with a previous study conducted by Elixhauser *et al*., which demonstrated the total cost of GC accounted for $1,800,000,000^19^. the total costs of either ECX followed by FOLFIRI or FOLFIRI followed by ECX was considerable less in our study.

Our results from the sensitivity analysis found that the utilities for PD state and PFS state were the most influential parameters, followed by transition probabilities in ECX arm and FOLFIRI arm. Of particular note, changes in costs of FOLFIRI had the greater impact on the results than combination of ECX costs. Furthermore, influence of cost of supportive drugs for FOLFIRI played an important role in the whole model. Since FOLFIRI regimen cost much more than ECX regimen ($2,275.46 versus $959.68), cost for PFS state was higher in FOLFIRI arm than in ECX arm, while cost for PD state was higher in ECX arm than in FOLFIRI arm. Additionally, more chemotherapy drugs are given by infusion in FOLFIRI combination, which were related with days in hospital and days if supportive drugs; while for ECX combination, treatment implement is more convenient because of the oral capecitabine. Thus, the costs of supportive drugs also showed a profound impact on the analysis model, which were related to patients’ length of stay, namely the longer the hospital stay, the more supportive drugs were administrated. The results were consistent with Nishimura *et al*., which suggested that the hospitals losses could be reduced by shortening the length of patients’ hospitalization^20^.

Indeed, our research may also carry potential drawbacks. First, the analysis was based on the information of the French group study, which was not a patient-level data. Nevertheless, our study embraces the limited information about the chemotherapy costs for AGC, and paves the road for further research in targeted agent combination treatments. Second, utilities for the state of PFS and PD were referenced with a previous published research^21^, which may influence the calculation of QALMs. Thus, data of EQ-5D form the AGC patients should have been collected to keep the accuracy. Third, the AE reported in the French group study were restricted because they simply dichotomized hematologic toxicity level into non-hematologic and hematologic. Thus, further randomized clinical trial data are needed to confirm the accuracy of AE cost.

To the best of our knowledge, this is the first cost-effectiveness analysis to determine the preferred strategy between FOLFIRI followed by ECX and ECX followed by FOLFIRI for AGC. Our results suggested ECX followed by FOLFIRI was a preferred strategy with more effectiveness and less cost compared with FOLFIRI followed by ECX. Hence, further phase III studies are warranted to confirm this difference and to develop the standard care for advanced gastric cancer.

## Materials and Methods

### Patients Characteristics

A French intergroup study enrolled patients in an open labeled, prospective, randomized phase III trial in 71 French centers. In this study, 416 patients with a median age of 61.4 years old were histologically confirmed with advanced gastric or esophago-gastric junction (EGJ) adenocarcinoma. Patients were randomized signed into two groups: ECX arm (ECX followed by FOLFIRI as first-/second-line therapy) or FOLFIRI arm (FOLFIRI followed by ECX as firs-/second-line therapy). The retrospective study was approved by medical ethics committee of West China Hospital, Sichuan University, People’s Republic of China, and we do not need to obtain written informed consent of participants. None of our authors directly interacted with any study participants.

### The treatments

The clinical trial has been described in the original publication [12]. In brief, for the ECX arm, epirubicinat at a dose of 50 mg/m^2^ was given by intravenous (IV) infusion in 15 minutes, meanwhile, cisplatin 60 mg/m^2^ was administrated though IV infusion in one hour on day one followed by oral capecitabine at a dose of 1 g/m^2^ twice a day from day 2 to day 15. The regimen was repeated every 3 weeks^22^. For FOLFIRI arm, irinotecan was given IV infusion at a dose of 180 mg/m^2^ in 90 minutes, and 400 mg/m^2^ leucovorin was given in two hours by IV infusion followed by fluorouracil at dose of 400 mg/m^2^ IV bolus and then fluorouracil 2,400 mg/m^2^ in 46 hours by continuous infusion, which was repeated every two weeks. The treatment was discontinued if patients encountered one of the following events: requested to dropout, disease progression, unacceptable toxicity or death. The treatment free interval for second-line treatment was at least three weeks until the recovery of biologic and clinical conditions. Tumor response was evaluated before the beginning of the treatment and repeated every 8 weeks until the disease progression.

### Clinical outcomes

In this French intergroup study cohort, the median TTP was 5.1 months in FOLFIRI arm compared with 4.1 months in ECX arm (p = 0.008). While, the median PFS and OS were 5.3 months and 9.5 months in ECX arm compared to 5.8 months and 9.7 months in FOLFIRI group, which were not significantly different. For the first-line treatment, the total grade 3/4 toxicity was 15.0% higher in ECX arm than in FOLFIRI arm (84.0% versus 69.0%, p < 0.001). In addition, the high-grade hematologic toxicity rate was 64.3% in ECX arm compared to 38.0% in FOLFIRI arm (p < 0.001). But the non-hematologic adverse events in ECX were similar to that in FOLFIRI arm (53.5 v 53.0%, p = 0.81).

### Overall concept of the Markov model

Treeage software (Treeage, Williamstown, MA, USA) was applied to build a Markov decision model evaluating the economic consequences and therapeutic efficacy associated with variable of interests and treatment strategies. The time horizon was 10 years, which was nearly life long and the transition cycle length was one month. The transitions diagram among model states is presented in Fig. 1. The costs were calculated from a Chinese societal perspective, and survival was reported in quality-adjusted life-months (QALMs). Incremental cost-effectiveness ratios (ICERs), defining as cost per unit of survival, was calculated as the difference in costs divided by the difference in effectiveness between a given strategy and the most cost-effective alternative.

### The strategies and Markov model structure

According to the profile of the French group study, two groups were analyzed: in ECX group, patients with AGC treated with ECX followed by FOLFIRI; FOLFIRI group, patients with AGC treated with FOLFIRI followed by ECX. Three clinical outcomes were examined: PFS, progressive disease (PD) and death (Fig. 1). Patients were assumed to enter the model at the PFS state, who received either ECX therapy or FOLFIRI treatment as the first-line regimens until the progressions of disease, intolerable toxicities or death. In the PD state, second-line treatment was predefined, namely, FOLFIRI for the ECX arm and ECX for the FOLFIRI arm. The transition probabilities of health states were estimated based on an equation used previously: *P (1 month)* = [*1* − (*0.5) ^ (1/median time to event*), which was derived from the equations below: *P* = *1* − *e*^*−R*^ *and R* = −*ln*[*0.5*]/(*time to event/number of treatment cycles*)^23^,^24^. Based on the equation, monthly transition probability from PFS state to PFS state (*p*PFS-PFS), from the PFS to progression disease (PD) (*p*PFS-PD), from PFS to death (*p*PFS-death), from PD to PD (*p*PD-PD) and from PD to death (*p*PD-death) were described in Table 3.

### The utilities

We calculated the cost-effectiveness in each group based upon the health-related QOL. Preference-based health states utility scores were derived from previously published studies and the values were set at 0.797 for PFS state, 0.6 for PD state and 0 for death state respectively^21^. Since the patients’ performance status and the treatment disposals in two groups were similar, the same utilities values were shared in this model.

### Measurement of costs

Costs were calculated from a Chinese societal perspective. Costs for the first-line therapies as well as second-line treatments were included in the analysis. Direct treatment related costs were taken into account, including costs of chemotherapy drugs, supportive care (mainly anti-emesis, hospitalization, grade 3–4 adverse events (AEs) related costs and necessary tests for efficacy and toxicities/safety evaluation during the treatments. Detailed data about the grade 3–4 AEs were derived from the records of the original study. All costs were converted into US dollars, with an exchange rate of $*1* = *¥6.39* (August 26^th^, 2015).

### Sensitivity analysis

One-way sensitivity analysis was performed to examine the impact of essential factors on our model, and the ranges of the factors analyzed were calculated by increasing or decreasing them by 30%^25^. According to World Health Organization (WHO) guidelines for cost-effective analysis, the willingness to pay (WTP) was set to $20,301.00 per year, namely $1691.75 per QALM, which was 3× GDP per capita of China in 2014^26^,^27^. In addition, probabilistic sensitivity analysis was performed by conducting a second-order Monte Carlo simulation to estimate different optimal strategies with varied WTP thresholds^25^, in which a cohort of 100,000 patients was simulated to imitate the process of AGC, and the model was run until all hypothetical patients died.

## Additional Information

**How to cite this article**: Wen, F. *et al*. Cost-effectiveness analysis of Fluorouracil, Leucovorin, and Irinotecan Versus Epirubicin, Cisplatin, and Capecitabine in patients with Advanced Gastric Adenocarcinoma. *Sci. Rep.* **6**, 36060; doi: 10.1038/srep36060 (2016).

**Publisher’s note:** Springer Nature remains neutral with regard to jurisdictional claims in published maps and institutional affiliations.

## Figures and Tables

**Table 1:** Results of the cost-effectiveness analysis.

	ECX	FOLFIRI
Cost ($)		
Costs for PFS per cycle		
Chemotherapy	959.68	2,275.46
Supportive drugs	652.42	1,223.30
Hospital	33.37	62.56
Test	173.29	173.29
AE	17.92	11.48
Total	1,836.68	3,746.09
Costs for PD per cycle		
Chemotherapy	2,275.46	959.68
Supportive drugs	1,223.30	652.42
Hospital	62.56	33.37
Test	173.29	173.29
AE	16.44	18.54
Total	3,751.04	1,837.30
Costs for PFS state	8,499.92	18,481.71
Costs for PD state	15,313.21	6,501.98
Total costs	23,813.13	24,983.70
Incremental costs		1,170.57
Effectiveness(QALMs)		
Effectiveness for PFS state	3.69	3.93
Effectiveness for PD state	2.45	2.12
Total effectiveness	6.14	6.06
Incremental effectiveness	0.08	
Cost/Effectiveness	3,879.72	4,125.84
Incremental cost/effectiveness		Dominated

AE, adverse event; PFS, progression-free survival; PD, progressive disease; QALM, quality-adjusted life month; ECX, epirubicin, cisplatin, and capecitabine; FOLFIRI, fluorouracil, leucovorin, and irinotecan.

**Table 2:** Adverse event costs of advanced gastric cancer patients treated with ECX or FOLFIRI per cycle per patient.

Grade 3 to 4	ECX (N)	FOLFIRI (N)	Cost for ECX ($)	Cost for FOLFIRI ($)
First-line	200	203		
nonhematologic	107	108	4.74	4.30
hematologic	129	78	13.18	7.18
total	—	—	17.92	11.48
Second-line	101	81		
nonhematologic	47	44	5.20	6.54
hematologic	44	35	11.24	12.00
total	—	—	16.44	18.54

ECX, epirubicin, cisplatin, and capecitabine; FOLFIRI, fluorouracil, leucovorin, and irinotecan; N, number.

**Table 3:** Transition probabilities and utilities used in the analysis.

	Value	Lower limit	Higher limit
Probabilities			
ECX group			
P_PFS-PFS_	0.81	0.56	1.00
P_PFS-PD_	0.12	0.09	0.16
P_PFS-death_	0.07	0.05	0.09
P_PD-PD_	0.85	0.59	1.00
P_PD-death_	0.15	0.11	0.20
FOLFIRI group			
P_PFS-PFS_	0.82	0.57	1.00
P_PFS-PD_	0.11	0.08	0.15
P_PFS-death_	0.07	0.05	0.09
P_PD-PD_	0.83	0.58	1.00
P_PD-death_	0.17	0.12	0.22
Utility			
PFS state	0.80	0.56	1.00
PD state	0.60	0.42	0.78
Death state	0.00	0.00	0.00

P: transition probability; PFS, progression-free survival; PD, progressive disease; ECX, epirubicin, cisplatin, and capecitabine; FOLFIRI, fluorouracil, leucovorin, and irinotecan.
